# A Dynamic Estimation of Service Level Based on Fuzzy Logic for Robustness in the Internet of Things

**DOI:** 10.3390/s18072190

**Published:** 2018-07-07

**Authors:** Bing Jia, Lifei Hao, Chuxuan Zhang, Dong Chen

**Affiliations:** 1 School of Computer Science, Inner Mongolia University, Hohhot 010021, China; jiabing@imu.edu.cn (B.J.); 31709063@mail.imu.edu.cn (L.H.); 31709075@mail.imu.edu.cn (C.Z.); 2 Inner Mongolia A.R. Key Laboratory of Wireless Networking and Mobile Computing, Hohhot 010021, China; 3 School of Computing and Information Sciences, Florida International University, 11200 S.W. 8th Street, Miami, FL 33199, USA

**Keywords:** Internet of Things, fuzzy logic, service level, estimation, changing rules

## Abstract

The Internet of things (IoT) technology is developing rapidly, and the IoT services are penetrating broadly into every aspect of people’s lives. As the large amount of services grows dramatically, how to discover and select the best services dynamically to satisfy the actual needs of users in the IoT service set, the elements of which have the same function, is an unavoidable issue. Therefore, for the robustness of the IoT system, evaluating the quality level of the IoT service to provide a reference for the users choosing the most appropriate service has become a hot topic. Most of the current methods just use some static data to evaluate the quality of the service and ignore the dynamic changing trend of the service performance. In this paper, an estimation mechanism for the quality level of the IoT service based on fuzzy logic is conducted to grade the quality of the service. Specifically, the comprehensive factors are taken into account according to the defined level changing rules and the effect of the service in the previous execution process, so that it can provide users with an effective reference. Experiments are carried out by using a simulated service set. It is shown that the proposed algorithm can estimate the quality level of the service more comprehensively and reasonably, which is evidently superior to the other two common methods, i.e., the estimating method by a Randomization Test (RT) and the estimating method by a Single Test in Steps (STS).

## 1. Introduction

The advent of ubiquitous wireless connectivity in conjunction with the ever-increasing deployment of pervasive computing technologies has changed the landscape of information and communication technologies. One of the most important examples is the Internet of Things (IoT) [1] metaphor, which is defined as “a world-wide network of interconnected objects uniquely addressable based on standard communication protocols”. IoT refers to the integration of large numbers of real-world objects, that is “things”, in the Internet and aims to simplify high-level interactions of the physical world to resemble interactions taking place in virtual electronic worlds. Theoretically, IoT can be applied to all kinds of domains, and IoT has been widely used in many key areas such as business, healthcare and industry [2], currently. For example, Berkers et al. proposed an ecosystem model around a smart, horizontal IoT service platform to realize IoT business [3]. Shang et al. discussed the application of IoT in e-commerce [4].

The IoT service has made great progress in practical applications, which has greatly facilitated our production and our life. As a large amount of services grows dramatically, how to discover and select the best services dynamically to satisfy the actual needs of users in the IoT service set, the elements of which have the same function, is an unavoidable issue. Therefore, evaluating the quality level of the service existing in the IoT to provide a reference for the users choosing the most appropriate service has become a hot topic in the current academic research. At present, researchers have not yet built a unified estimating method for the quality level of the IoT service, most of which refer to the evaluation system of web service, i.e., Quality of Service (QoS), which is the most widely-used non-functional measurement standard, and Quality of Experience (QoE), which is an evaluation method based on the degree of user acceptance, including both the subjective and objective aspects. For example, in [5], a QoS ontology was proposed to describe the contextual features of the IoT services embedded in physical entities. In [6], a multi-dimensional user requirement QoS model was used to describe service oriented toward physical objects. In [7], four kinds of QoS calculation models were proposed, which can be used to decompose and optimize the complex QoS requirements of IoT services. Aazam et al. devised a QoE-based resource estimation in IoT on the basis of the Relinquish Rate (RR) to enhance QoS [8]. The method reported in [9] adopted multiple linear regression analysis to evaluate QoE in IoT. In [10], a framework was given of scalable QoE modeling based on the massive amount of quality metrics for explosively increasing applications in IoT. Another IoT service measurement approach is to establish a Service Level Agreement (SLA) or its variant [11,12,13] between the network service providers and the users. The SLA is in general a contract that defines some terms such as the type of service, the quality of service and the payment of the customer, and its application in IoT has just begun, with many drawbacks, though it had been studied for many years in web service [11]. All these research works above just use some static data to evaluate the quality of the service and ignore the dynamic changing trend of the service performance during the testing or executing process. For example, most evaluation methods based on QoS use the static service parameters to determine the service level, with the level of services remaining unchanged after evaluations. Most of the methods based on QoE rely too much on users’ subjective feelings, and the evaluation result is uncertain due to the differences among each one’s feeling. In addition, the traditional approaches to handle Service Level Agreements (SLAs) are limited to a predefined service quality provided for a fixed price. Likewise, SLA provides only a static QoS description, and the modification of the predefined QoS parameter results in a re-negotiation, usually combined with a service termination, which takes plenty of time and resources [13]. However, the status, or more precisely, the properties of services change over time moment by moment, such as the executing efficiency, the reliability, the availability, etc. When service’s efficiency, reliability or availability decrease or increase, the service level evaluated must reflect the latest status of services as soon as possible. Dynamic estimation of service level, therefore, is very necessary, such as considering the latest service execution time to measure the property of its efficiency and the history of service execution accuracy to measure its reliability by giving different weights to different periods of time. Combined with these factors, the rangeability of the level estimated can be influenced appropriately in order to achieve the effect of dynamic evaluation.

On the other hand, IoT services enable the interconnection of a large number of smart devices (things) using a combination of networks and computing technologies. However, an influx of interconnected things makes a greater demand on the underlying communication networks and affects the implementation effect of the service [14]. All of these make the performance of the service fluctuant, which may lead to a reduced availability of the service selection. Some research works have been carried out to improve the availability. For example, in [15], a QoS architecture for IoT that focused on the control-mechanism for transferring and translation of QoS was proposed to improve the QoS of IoT. In [16], a new and trustable framework for a Mobile Edge Computing (MEC) management/orchestration system with crucial security and authentication components by which it ensures the delivery of users’ QoE was given. Furthermore, in [17], a cooperative evolution approach for service composition under the restrictions of QoS was proposed to address the aim of distinguishing prospective services out of many “similar” services and identifying needed services with regard to the criteria of QoS. Because locating and invoking suitable services are quite challenging and traditional service discovery and selection approaches have been proven inadequate, Karageorgos et al. proposed a decentralized service discovery and selection model [18] based on Artificial Potential Fields (APFs), which are formed on each user service request and become active at points where services can be provided. However, they did not consider some cases such as multiple variables in generating artificial potential fields, etc. In practical industrial applications, many scholars also have put forward various methods to solve the problem of the selection strategy for IoT services. For instance, the authors of [19] proposed a register service selection-based security architecture to get rid of these problems in oil production materials, pharmaceuticals and compound process industries. However, their approach only can be applied to some specific areas, not as a generic method to solve the IoT service selection problem in all kinds of areas.

Based on the above analysis, we can conclude that one effective way to solve the problem of IoT service selection in IoT is to grade the services, as well as the similar problem in other domains [20]. It can achieve the purpose of filtering poor quality services to ensure the quality of services and developing the optimal selection strategy by evaluating the efficiency, reliability and other comprehensive indexes of services. Nevertheless, there is no specific method for rating or estimating IoT services [21] or grading the level of services only by a very simple method such as the estimating method by a Randomization Test (RT) and the estimating method by a Single Test in Steps (STS) [22]. Neither of these methods consider the angular and the accidental factors, so that the resulting service level is not very reasonable, and it is difficult to achieve the purpose of filtering out bad services and preserving the quality services. For example, Kim et al. have studied the development and application of a taxonomy for IoT services [23], but they did not propose a specific level evaluation method.

In this paper, a dynamic evaluation method is presented to estimate the quality level of the IoT service, which takes into account the comprehensive and changeable factors, and each new evaluation can achieve a higher level for better service indicators or vice versa, i.e., dynamic. Specifically, an estimation mechanism for the quality level of the IoT service based on fuzzy logic ([24] also proved fuzzy theory can be applied to IoT effectively) is used to grade the quality of the service, which only considers the implementation effect of the service in the previous execution process, instead of considering what kind of evaluation system is adopted, which ensures the proposed method as a relatively generic approach. Firstly, a series of level changing rules based on fuzzy logic is defined, which can be used to calculate the quality level of the service by testing the implementation effect of the service. Furthermore, the dynamic influence factor for the rangeability of the fuzzy membership degree is analyzed and formulized. Experiments are carried out by using a simulated service set and show that the performance of the proposed method can achieve a more stable result and is more in line with the actual service level in comparison with existing approaches, i.e., RT and STS. To sum up, our main contribution is that the proposed method, which fully considers the dynamic changing of the service and intensively takes the uncertainty of QoS into account, i.e., combining the theory of fuzzy logic, is a relatively better approach in terms of appropriate evaluation of IoT services compared to the other methods. At the same time, it can be used as a general method, since it does not only limit the specific IoT sub-domain of the service, but also extracts the more common properties in the IoT service for dynamic evaluation, so that it can be applied to a specific domain by adding or deleting some certain attributes.

The rest of the paper is organized as follows. Section 2 discusses the basic theory of fuzzy logic and proposes the estimation method for IoT service level based on fuzzy logic, as well as gives some cases to illustrate it. In Section 3, the service execution time and service reliability as the evaluation parameters are added to the influence factor for the rangeability of the fuzzy membership degree; their definition is given, and how they change the amplitude of the membership degree is explained. Section 4 establishes a probabilistic model to express the service passing the test or not and reports the experimental results and performance analysis in comparison with the existing approaches. Finally, Section 5 concludes our work and sheds light on future works.

## 2. An Estimation Mechanism for the Quality Level of the Service Level Based on Fuzzy Logic

In 1965, American scholar Zadeh put forward a method to describe fuzzy phenomena in mathematics: fuzzy sets. The fuzzy set theory holds that the object in the domain of discourse becomes gradually transitive due to the nature of the collection, rather than having a sudden change. The appropriate membership function is established, and the fuzzy objects can be analyzed by the relevant operation and transformation of fuzzy sets [25]. The fuzzy logic extends the binary logic zero and one to the closed interval [0,1] for any value in it, and it can be consecutive with an infinite number of values, which belongs to the interval [0,1]. In domain of discourse *U*, there is a given mapping μ:*U* → [0,1]; this means μ determines a fuzzy set on *U*, marked as $\tilde{c}$. μ is called the membership function of $\tilde{c}$’, and this is denoted as $\mu_{\tilde{c}}$. It represents the degree to which an object belongs to the fuzzy set [26]. When $\mu_{\tilde{c}}\left(u\right)=1$, *u* completely belongs to the fuzzy set $\tilde{c}$, and *u* completely does not belong to fuzzy set $\tilde{c}$ when it is equal to zero.

The service level reflects the comprehensive ability of the service, i.e., the overall service implementation and the evaluation of the service by the users (or the rating agency). The initialization of the service level can be evaluated by testing. The precondition of the testing is to have a set of evaluation samples based on a certain standard, and the evaluation sample is divided into the service levels. A simpler assessment (i.e., STS) is that if the service passes a test of an evaluation sample of *n*, we speak about the service reaching a level of *n*. This method has great randomness such that the sample of the same rating may not be able to measure the same angle, i.e., the service could only pass the evaluation testing on these angles and cannot through testing at other angles. As a result, we need to do a series of tests to evaluate the level of service. In this paper, a method based on fuzzy logic is proposed to estimate the service level, and we can find out which level the service is most likely to reach based on the evaluation test.

For the sake of illustration, this paper divides the service into six levels (of course, it can be extended to *n* levels), and we use *H* to represent it, so H={1,2,3,4,5,6}. The membership degree of each level is denoted by $\mu_{k}$(i), i=1…6. We use *K* to represent the fuzzy set of the service level, and *K* is expressed as: K={i|$\mu_{k}\left(i\right)$, *i*∈*H*}, where 0 ≤ $\mu_{k}\left(i\right)$ ≤ 1, $\Sigma_{i=1}^{6}$ $\mu_{k}\left(i\right)$ ≤ 1. For a service resource, the highest degree of membership is the level of the service resource.

For example, assume the membership of the six levels of a service after testing is {0.1,0.4,0.2,0.1,0.4,0}, so the fuzzy set of the service level can be denoted as K={(1,0.1),(2,0.4),(3,0.2),(4,0.2),(5,0.1),(6,0)}. As can be seen from the set *K*, the membership degree of Level 2 is the highest, so it can be estimated that the service is most likely to reach Level 2.

Based on the fuzzy logic proposed in [27], the membership grade changing rules of fuzzy set *K* are given in Table 1. *m* is the grade of the evaluation sample; i=1…6. *k* is the number of rounds currently being calculated; and *q* is defined as the changing factor of the membership degree, which affects the changing rangeability of the membership degree, i.e., the higher *q* is, the more the membership degree $\mu_{k}\left(i\right)$ of the current level increases and vice versa.

The rule is defined in the way given below when it is used in practice. When the service passes the evaluation testing at a certain level, this level and the following level adopt the increase rule, while the above levels adopt the decrease rule. For cases that do not pass, the membership grade does not change [27]. For example, as shown in Table 2, the parameter *q* is set to 0.4 in order to explain the changing rules of the membership grade in *K*.

From this table, we can doubtlessly deduce that the service should be at the second level. Though it passed the third test, the fourth test at the same level failed. It is assumed that the last time the service was tested, it happened to coincide with the evaluation angle of the evaluation sample, which led to the passing of the test; or in this testing, the evaluation angle of the evaluation sample did not coincide with the test, so that the test failed. When performing the fifth service test at the same level, it failed again. It is possible to have a higher probability of not reaching this level, then lowering the level to continue the test. With this algorithm, some interference information can be excluded (i.e., some angle, contingency factor, etc.) to make the result more reasonable [27]. The level of the service can be evaluated by a certain number of tests.

In the same test, the degree of membership change obtained is shown in Table 3, after each stage when q=0.6.

At this point, we can find that the membership grade of Service Level 3 is the highest in the third test. If the test has finished at this time, the level of the service will return to 3. Therefore, the accuracy of the service level estimation depends on the number of tests and the strategy of testing. In general, the test at the same level should be repeated several times, especially for the case of failing several times for a certain level of test, and the level must be reduced to test again. For example, in Table 3, the test level was reduced to 2 when the forth and fifth test of Level 3 were not passed.

In addition, in the event of two consecutive tests of the service not passing a certain level and still not passing the test after reducing the level the first time, we will make it equal to the first failure to try the level. In this case, we can guarantee the same subproblems, so that it is convenient for us to implement this using the iteration method.

Finally, considering the boundary conditions, the test level is no longer reduced when the test level is 1, and the results of the fuzzy set are integrated with the other level test for the test at Level 6 due to the two consecutive calculations showing a rising rule.

## 3. The Influence Factor for the Rangeability of the Fuzzy Membership Degree

Based on the above two examples, we can clearly see that parameter *q* determines the rangeability of the membership degree. Decrease rules, for example, Δ = $(\mu_{k-1}\left(i+1\right)-\mu_{k-1}\left(i\right))q$ with the variation of $\mu_{k}\left(i\right)$. Since the service level is a comprehensive evaluation of the service, parameter q can consider many changeable factors, such as the speed of the test completed for services, the reliability, satisfaction and interest of the samples, etc. All of these factors may change over time, and the changed factors (better or worse) will affect the *q* in turn (higher or lower). Therefore, the next evaluation can produce a different result, which reflects the dynamic nature of the proposed method. In this paper, we selected two changeable factors to illustrate, i.e., the execution time of the service test and the reliability of the sample to illustrate.

First of all, the speed of service execution is compared with the similar services by a regular execution time and the longest execution time. The service completes the test in a time less than or equal to the regular execution time, indicating that the service response performance is very good; the performance of service response is flat when the service execution time is between regular and maximum time; if it exceeds the maximum time, we think the service response performance is poor [27]. The service completion test speed factor can be defined as Definition 1 in the form of a function to express.

**Definition** **1.** *$T_{a}$ is the regular time to complete the evaluation sample test for the service; $T_{b}$ is the maximum time to complete the test for the service; ρ is the adjustment coefficient, 0<ρ<1. The function F(t) that denotes the speed of testing a service is defined as follows (for each evaluation sample test, the values of $T_{a}$ and $T_{b}$ can be different):*

(1) $$F\left(t\right)=\left\{\begin{array}{ccc} 1 & \; & if\;t\leq T_{a}\\ 1-\rho \times (\frac{t-T_{a}}{T_{b}-T_{a}}) & \; & if\;T_{a}<t<T_{b}\\ 0 & \; & if\;t\geq T_{b} \end{array}\right.$$

Secondly, RE is used to represent the reliability of samples, 0<RE<1. The larger the value of RE is, the more reliable the evaluation sample is and the faster the service level needs to change. The reliability of the evaluation sample reflects the historical accuracy of the sample. According to different historical stages, we have given different adjusting parameters for the calculation of its accuracy to reflect the reference value of the accuracy of different historical periods. Generally speaking, the most recent data are the most valuable, so we can obtain Definition 2 for RE and $\theta_{1}<\theta_{2}<\theta_{3}$ in most cases.

**Definition** **2.** *Set up $T_{1}$ and $T_{2}$ as two historical moments. $T_{2}$ is a closer moment to the present. Therefore, there are three time periods, the time period before $T_{1}$, the time period $T_{1}$–$T_{2}$ and the time period $T_{2}$ to present. $\theta_{1}$, $\theta_{2}$, $\theta_{3}$ have been used as factors to adjust the correct reference weight of the three historical periods. Each of these three factors is greater than zero and less than one, and $\theta_{1}+\theta_{2}+\theta_{3}=1$. $RE_{1}$, $RE_{2}$ and $RE_{3}$ represent the accuracy of these three historical periods, so we have:*

(2) $$RE=\theta_{1}\times RE_{1}+\theta_{2}\times RE_{2}+\theta_{3}\times RE_{3}$$

If we use $correct_{1}$, $correct_{2}$, $correct_{3}$ respectively representing the same number of services evaluated by sample testing and the last evaluation results in three historical periods, $test_{1}$, $test_{2}$, $test_{3}$, respectively representing the total number of service tests evaluated by the sample in the three historical periods, therefore we have:

(3) $$RE_{i}=\frac{Correct_{i}}{test_{i}}\;i=1,2,3$$

Finally, according to the characteristics of *q*, set:

(4) $$q=\sigma \times RE^{c}\times F\left(t\right)$$

where “*c*” is a constant, 0<c<1. “σ” is an adjustable coefficient to reflect the difference in the evaluation of different services. F(t)=1 when the service test execution time is within the normal range, and q becomes larger as the RE becomes higher. F(t) becomes smaller when the test time goes beyond the normal range, and *q* becomes smaller as RE becomes lower. F(t)=0, and q=0 when the test time exceeds the maximum time, indicating the failure of the membership changing rule [27].

In particular, this mechanism can be extended to achieve a level of self-renewal in service-providing applications; because when introducing service level matching in the previous section, we allowed the service to be run across level calling, which is consistent with the strategy of the evaluation test. In addition, the parameter *q* that determines the degree of membership changing is evolved according to the user’s feedback on the performance of the service such as the user’s satisfaction degree, interest degree, etc. At this time, the parameter RE can be understood as the credibility of user evaluation.

## 4. Experiment

In this section, we will carry out the simulation experiment by programming according to the above method in order to determine the various parameters in the formulas above and to compare the the practical effects on evaluating levels, as well as the execution efficiency between our Fuzzy Logic Estimating Level (FLEL) mechanism and the other two methods on the test service set. The changing rules for the membership degree adopted are as shown in Table 1.

In Section 4.1, a reasonable range of q values is determined by defining the probability model that the service actually passes the test and comparing the average service level estimated by the FLEL mechanism with the increase of the q value. Based on this, the parameters ρ in Formula (1) and σ in Formula (4) are determined, and a test service set containing 300 simulated services is generated to be used by the three methods in the following section. Finally, the evaluation processes of the other two methods that will be compared are described.

In Section 4.2, the actual effects of three methods on estimating the service level are compared to the average estimation level result and its variance, and the executing efficiency by the average passing time.

### 4.1. Setup

#### 4.1.1. Parameters

To simulate the actual probability of service at a certain level, we define the probability model of a service passing a certain level as Formula (5), where *n* is the largest level, α is the basis of probability and β is the factor for the probability decreasing. In this experiment, we take *n* as 6, α as 0.4 and β as 0.1, which are more consistent with the actual situation of the service passing the tests.

(5) $$Prob_{pass}=\alpha +\left(n+1-level_{test}\right)\times \beta$$

**Figure 1:**
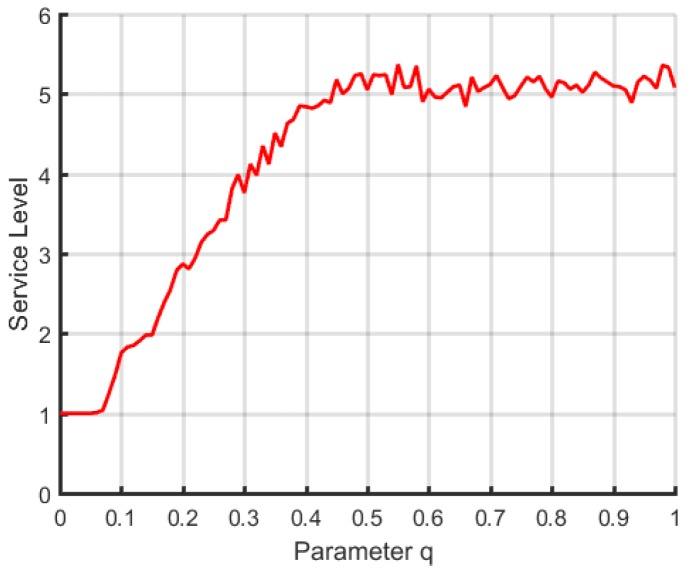
The changing trend of the average value of the service level estimated with the increasing of the *q* value.

On the basis of this probability model, we test the changing of the estimated level with the increasing of the *q* value after the implementation of service level estimation based on fuzzy logic, where the ordinate numeral denotes the average result of estimating 100 times, as shown in Figure 1.

Through the above figure, we can conclude that the estimated results nearly had no change after the *q* value reached around 0.5. At the same time, the estimated level change was relatively flat from a *q* value of 0.5–1.0. Therefore, we need to adjust the ρ in Formula (1) and the σ in Formula (4) for the final calculation of the *q* value to be distributed more homogeneously between 0.1 and 0.5.

#### 4.1.2. Service Set

In order to test the actual effect of this method, we randomly generated a test set of 300 services and randomly divided each service into one of four categories, as partly shown in Table 4. The value range of the current service’s test execution time is [1,120], and we set $T_{a}$ as 60 and $T_{b}$ as 100 according to Definition 1. The scope of the credibility evaluation sample parameters $test_{1}$, $test_{2}$, $test_{3}$ is set to [1,100]; the scope of $correct_{1}$, $correct_{2}$, $correct_{3}$ is respectively set to $\left[1,test_{a}\right]$ $\left[1,test_{b}\right]$ $\left[1,test_{c}\right]$; and the parameters $\theta_{1}$, $theta_{2}$, $theta_{3}$ are 0.2,0.3,0.5 respectively, according to Definition 2. Through many attempts, the final setting of the value of ρ is 0.8, and σ is set between [0.6,0.75] based on the service type so as to reflect the difference in the evaluations of different services.

**Figure 2:**
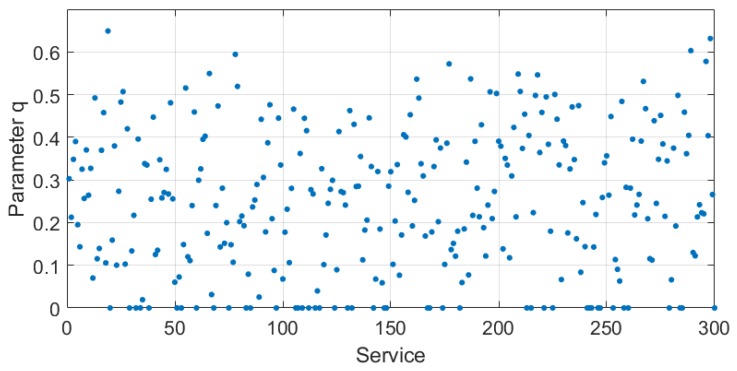
Parameter *q* distribution of 300 services.

Combining Formulas (1), (2) and (4), the *q* value distribution of 300 services is shown in Figure 2 according to the above parameters.

We can arrive at the conclusion that the *q* value of most services was evenly distributed between 0.1 and 0.5 for all the services that were randomly generated except for the q value of the service with an execution time exceeding the longest response time (i.e., $t>T_{b}$), which is 0. There are only 18 services with a *q* value of more than 0.5, which means that 94% of the services have a good parameter for the membership degree changing rangeability.

#### 4.1.3. The Other Two Methods

**Figure 3:**
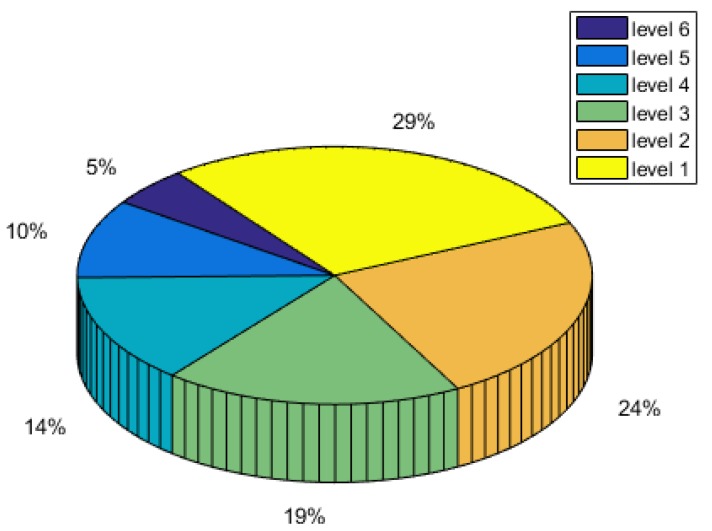
Roulette probability model.

Estimating method by the Randomization Test (RT): This method finds the highest level ratio by comparing each level ratio of the evaluated service and regarding it as the final evaluation result. We use the roulette probability model to simulate the passing probability of the service by this method due to the difference between this method and our FLEL method on evaluation levels. The probability model is shown in Figure 3. This method also can be regarded as testing for every level randomly and takes the maximum passing rate level as the final result after several rounds of testing.

Estimating method by the Single Test in Steps (STS). This method is the same as the FLEL method for the service level estimation process, but it increases the test level only after a single successful test, as well as drops the test level only after a single test failure. We adopted the same probability model, as FLEL passed the service level test for this approach, as shown in Formula (5).

### 4.2. Results and Analysis

#### 4.2.1. Estimation Result

We compared the proposed FLEL mechanism with the other two methods for the actual estimation results on the test service set, and the degree and dispersion of the level result estimated by the three methods can be assessed by the mean value and the variance of the service level obtained by experiment. We expect to get a more moderate level average (i.e., not too high or not too low), as well a larger variance to ensure the discrete degree of the estimated results, which will maximize the differences between services.

**Figure 4:**
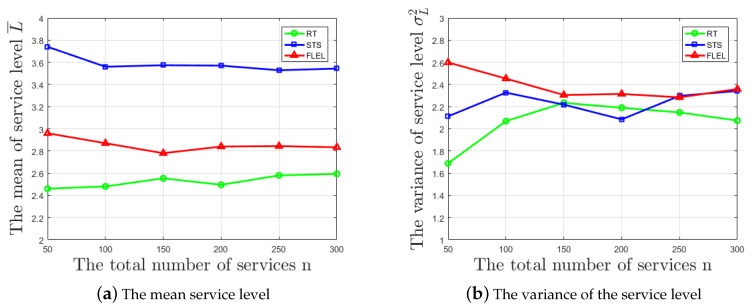
The changing of the service level with respect to different amounts of service. RT, Randomization Test; STS, Single Test in Steps.

Formula (6) defines the mean value of estimating service level $\bar{L}$, where $L_{i}$ represents the estimation level of the *i*-th service and *n* represents the total number of services. Formula (7) defines the variance of estimating service level $\sigma_{L}^{2}$, where $L_{i}$ and *n* are the same as above. Finally, as the total number of services *n* increases, the changing of $\bar{L}$ and $\sigma_{L}^{2}$ is plotted in Figure 4a,b, respectively.

(6) $$\bar{L}=\frac{\sum Li}{n}\;i=1\ldots n$$

(7) $$\sigma_{L}^{2}=\frac{{\left(L_{i}-\bar{L}\right)}^{2}}{n}\;i=1\ldots n$$

It can be seen that RT has a lower estimation result mean value of service level, and STS’s result is higher, while the result of the FLEL mechanism is relatively moderate, as shown in Figure 4a, which means FLEL has a better estimation result according to the discussion above.

Moreover, as can be seen from Figure 4b, FLEL always has the highest variance compared with the other two methods, so that its estimation results of the service level are more discrete. In another words, it can fully reflect the differences of quality between each service, which is more convenient for providing an effective reference to users.

Furthermore, combing the result of the discussion in Section 2, it can also be shown that the FLEL mechanism can eliminate interference information such as perspective and accidental factors, etc., which makes the evaluation better conform to the actual situation of the service, while the other two methods do not consider these factors.

To sum up, based on the above discussion, the proposed mechanism FLEL shows a better effect than the other two methods (RT and STS) in estimating the service level.

#### 4.2.2. Efficiency Measure

As stated earlier, the FLEL method’s estimated result of the service level is more accurate and objective, which depends on its strategy and the number of tests to a certain extent. Therefore, does that means the FLEL method’s efficiency is low and that it will greatly extend the time consumption of the evaluation?

In this section, we compare the efficiency of the three methods by using the Average Passed Times (APTs).

For RT, APT mainly depends on the number of rounds tested at six levels so that $APT_{RT}=6\times n$, where *n* is the number of rounds tested, as mentioned in Section 4.1.3.

For the STS, APT is equal to the result level, namely $APT_{STS}=Level_{service}$, which is a constant value.

**Figure 5:**
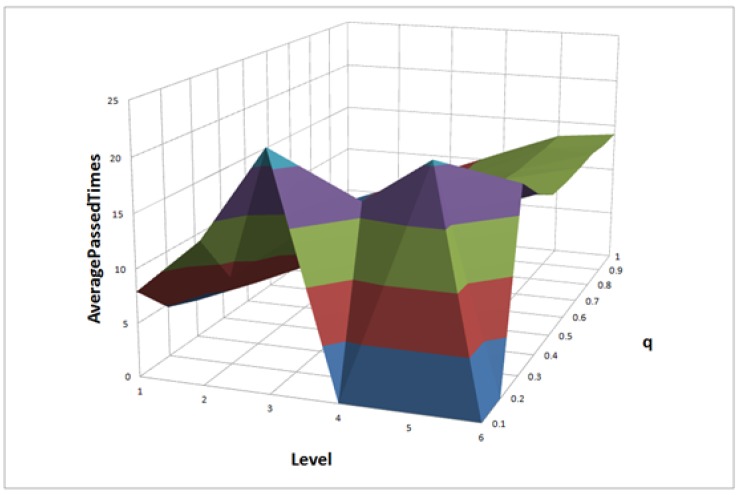
The Average Passed Times (APTs) of FLEL.

For FLEL, we can only get the APT through the actual test. Accordingly, for each *q* value from 0.1–1.0, we calculate 1000 times to get the APT of each level, as shown in Figure 5. We can find that the maximum APT is about 22 when *q* is 0.1 and the service level is evaluated at Level 3. Besides, in the sixty ranking results, 91.7% of APTs are less than 15. This can be approximated to $APT_{FLEL}\leq 15$, which is a constant value.

As a result, $APT_{STS}<APT_{FLEL}<APT_{RT}$.

Through the above analysis, the APTs of FLEL and STS are smaller than the APT of RT, meanwhile the APTs of FLEL and STS are constant in the same order of magnitude. We can obtain that the efficiency of FLEL is very close to the traditional methods or even higher, and its evaluation is more stable and more in line with the actual level of service.

## 5. Conclusions

In the application of IoT services, one effective way to discover the service needed by users and to develop a service selection strategy is to grade the services. Due to the imperfect evaluation or estimation methods for services, this paper proposed a dynamic IoT service level estimation method based on fuzzy logic. On account of fuzzy set theory, this method sets reasonable membership grade changing rules and takes into account the comprehensive factors, which are both changeable and generic, such as service execution efficiency and service reliability. In the experimental section, we determined the various parameters of services according to the distributed range of the q value first and then generated the service test set, which conformed to the actual situation. By comparison with two classical estimation methods, the experimental results showed that the FLEL method has a suitable level result, and it can maximize the quality differences between services when evaluating the level of service. It was proven that the efficiency of this method is close to the other methods or even higher through the comparison of APTs. Our future work will include optimizing the membership grade changing rules by combining with a new theory, further expanding the scale of the experiment to ensure the stability of this method and applying this method in practice to examine its actual effect, e.g., adding a lightweight self-renewal module of the service level based on the proposed mechanism to the actual service in the IoT circumstance, so that the service level can change dynamically through the service itself instead of through the users, which can address the issue of service selection in a more efficient way.

## Figures and Tables

**Table 1:** Membership grade changing rules.

Condition	Rule	Type
*i* < *m*	$\mu_{k}$(*i*) = $\mu_{k-1}$(*i*) − $\mu_{k-1}$(*i*)*q* + $\mu_{k-1}$(*i* − 1)*q*	Increase rule
*i* = *m*	$\mu_{k}$(*i*) = $\mu_{k-1}$(*i*) + $\mu_{k-1}$(*i* − 1)*q* + $\mu_{k-1}$(*i* + 1)*q*	Increase rule
*i* > *m*	$\mu_{k}$(*i*) = $\mu_{k-1}$(*i*) − $\mu_{k-1}$(*i*)*q* + $\mu_{k-1}$(*i* + 1)*q*	Decrease rule

**Table 2:** An example of the membership grade changing rule when q=0.4.

Initial μ = {1, 0, 0, 0, 0, 0}	q=0.4
1 μ = {0.6, 0.4, 0, 0, 0, 0}	Pass Level 2
2 μ = {0.36, 0.64, 0, 0, 0, 0}	Pass Level 2
3 μ = {0.216, 0.528, 0.256, 0, 0, 0}	Pass Level 3
4 μ = {0.216, 0.528, 0.256, 0, 0, 0}	Do not pass Level 3
5 μ = {0.216, 0.528, 0.256, 0, 0, 0}	Do not pass Level 3
6 μ = {0.1296, 0.7168, 0.1536, 0, 0, 0}	Pass Level 2

**Table 3:** The example of the membership change rule when q=0.6.

Initial μ = {1, 0, 0, 0, 0, 0}	q=0.6
1 μ = {0.4, 0.6, 0, 0, 0, 0}	Pass Level 2
2 μ = {0.16, 0.84, 0, 0, 0, 0}	Pass Level 2
3 μ = {0.064, 0.432, 0.504, 0, 0, 0}	Pass Level 3
4 μ = {0.064, 0.432, 0.504, 0, 0, 0}	Do not pass Level 3
5 μ = {0.064, 0.432, 0.504, 0, 0, 0}	Do not pass Level 3
6 μ = {0.0256, 0.7728, 0.2016, 0, 0, 0}	Pass Level 2

**Table 4:** Part of the test service set.

Service No.	Type	t	${\mathrm{Test}}_{1}$	${\mathrm{Test}}_{2}$	${\mathrm{Test}}_{3}$	${\mathrm{Correct}}_{1}$	${\mathrm{Correct}}_{2}$	${\mathrm{Correct}}_{3}$
Service 131	Type 2	75	44	53	72	44	43	46
Service 132	Type 3	116	60	35	63	1	32	44
Service 133	Type 3	46	12	77	2	2	1	2
Service 134	Type 4	58	38	45	54	14	4	6
Service 135	Type 1	35	43	83	6	3	57	2
Service 136	Type 2	50	89	62	75	49	15	1
Service 137	Type 4	81	59	68	16	16	13	12
Service 138	Type 4	21	83	91	54	77	67	36
Service 139	Type 3	104	11	23	43	10	7	22
Service 140	Type 2	23	77	85	37	41	13	14

## References

[1] Atzori L., Iera A., Morabito G. (2010). The Internet of Things: A survey. Comput. Netw..

[2] Munteanu L., Dumitriu D., Chiroiu V., Marin D. (2015). On the tactile sensing based on the smart materials. Comput. Mater. Cont..

[3] Berkers F., Roelands M., Bomhof F. Constructing a multi-sided business model for a smart horizontal IoT service platform. Proceedings of the 2013 17th International Conference on Intelligence in Next Generation Networks (ICIN).

[4] Shang X., Zhang R., Chen Y. (2012). Internet of Things IoT Service Architecture and its Application in E-Commerce. J. Electr. Commer. Organ..

[5] Li L., Liu N., Li G. (2016). Method to QoS-based dynamic service composition in semantic Web of Things. Appl. Res. Comput..

[6] Zhang T., Zhang X., Liu Z.D. (2013). Requirement-driven service composition approach for Internet of Things. Appl. Res. Comput..

[7] Zhou M., Yan M.A. (2013). QoS-aware computational method for IoT composite service. J. China Univ. Posts Telecommun..

[8] Aazam M., St-Hilaire M., Lung C.H., Lambadaris I. MeFoRE: QoE based resource estimation at fog to enhance QoS in IoT. Proceedings of the International Conference on Telecommunications.

[9] Li L., Rong M., Zhang G. An Internet of Things QoE evaluation method based on multiple linear regression analysis. Proceedings of the International Conference on Computer Science & Education.

[10] Ikeda Y., Kouno S., Shiozu A., Noritake K. A framework of scalable QoE modeling for application explosion in the Internet of Things. Proceedings of the 2016 IEEE 3rd World Forum on Internet of Things (WF-IoT).

[11] Gaillard G., Barthel D., Theoleyre F., Valois F. (2014). SLA Specification for IoT Operation—The WSN-SLA Framework. Netw. Int. Archit..

[12] Singh A., Viniotis Y. An SLA-based resource allocation for IoT applications in cloud environments. Proceedings of the 2016 Cloudification of the Internet of Things (CIoT).

[13] Grubitzsch P., Braun I., Fichtl H., Springer T., Hara T., Schill A. ML-SLA: Multi-Level Service Level Agreements for Highly Flexible IoT Services. Proceedings of the 2017 IEEE International Congress on Internet of Things (ICIOT).

[14] Awan I., Younas M., Naveed W. Modelling QoS in IoT Applications. Proceedings of the International Conference on Network-Based Information Systems.

[15] Duan R., Chen X., Xing T. A QoS Architecture for IOT. Proceedings of the CPSCom 2011: The 4th IEEE International Conference on Cyber, Physical, and Social Computing.

[16] Tahaei H., Ko K., Seo W., Joo S. (2018). A QoE Based Trustable SDN Framework for IoT Devices in Mobile Edge Computing. Advances in Computer Science and Ubiquitous Computing.

[17] Liu J., Chen Y., Chen X. (2013). A Cooperative Evolution for QoS-driven IoT Service Composition. Automatika.

[18] Karageorgos A., Karageorgos A., Houstis C. (2017). Decentralized service discovery and selection in Internet of Things applications based on artificial potential fields. Serv. Oriented Comput. Appl..

[19] Kanagaraju P., Nallusamy R. (2018). Registry service selection based secured Internet of Things with imperative control for industrial applications. Clust. Comput..

[20] Yildizel S.A., Öztürk A.U. (2015). A study on the estimation of prefabricated glass fiber reinforced concrete panel strength values with an artificial neural network model. Comput. Mater. Cont..

[21] Andročec D. (2017). Overcoming Service-Level Interoperability Challenges of the IoT. Computer Communications and Networks.

[22] Khalil A., Mbarek N., Togni O. Service Level Guarantee Framework for IoT environments. Proceedings of the International Conference on Internet of Things and Machine Learning.

[23] Kim E.A., Kim K.S., Leem C.S., Lee C.H. (2015). A Study on Development and Application of Taxonomy of Internet of Things Service. J. Soc. e-Bus. Stud..

[24] Kaur J., Kaur K. (2015). A fuzzy approach for an IoT-based automated employee performance appraisal. Comput. Mater. Cont..

[25] Zadeh L.A. (1965). Fuzzy sets. Inf. Control.

[26] Zadeh L.A. (1978). Fuzzy sets as a basis for a theory of possibility. Fuzzy Sets Syst..

[27] Zhuang Z. (2007). Estimation method of student’s cognitive level in self-adaptive distance tutoring system. Comput. Eng. Appl..

